# Synergistic Catalysis of Water-Soluble Exogenous Catalysts and Reservoir Minerals during the Aquathermolysis of Heavy Oil

**DOI:** 10.3390/molecules29163761

**Published:** 2024-08-08

**Authors:** Qian Wang, Shu Zhang, Xiang Chen, Jianjun Ni, Jialu Du, Yongfei Li, Xin Xin, Bin Zhao, Gang Chen

**Affiliations:** 1 Shaanxi University Engineering Research Center of Oil and Gas Field Chemistry, Xi’an Shiyou University, Xi’an 710065, China; 2 Shaanxi Province Key Laboratory of Environmental Pollution Control and Reservoir Protection Technology of Oilfields, Xi’an Shiyou University, Xi’an 710065, China; 3 Xi’an Petroleum Great Petroleum Technology Co., Ltd., Xi’an 710075, China; 4 Xi’an Changqing Chemical Group Co., Ltd. of Changqing Oilfield, PetroChina Oilfield Company, Xi’an 710200, China; 5 Department of Crop Soil Sciences, Washington State University, Pullman, WC 99163, USA; 6 Department of Statistics, North Dakota State University, Fargo, ND 58102, USA

**Keywords:** water-soluble, catalytic cracking, heavy oil, mechanism

## Abstract

Oil serves as the essential fuel and economic foundation of contemporary industry. However, the use of traditional light crude oil has exceeded its supply, making it challenging to meet the energy needs of humanity. Consequently, the extraction of heavy oil has become crucial in addressing this demand. This research focuses on the synthesis of several water-soluble catalysts that can work along with reservoir minerals to catalyze the hydrothermal cracking process of heavy oil. The goal is to effectively reduce the viscosity of heavy oil and lower the cost of its extraction. Based on the experimental findings, it was observed that when oil sample 1 underwent hydrothermal cracking at a temperature of 180 °C for a duration of 4 h, the amount of water added and catalyst used were 30% and 0.2% of the oil sample dosage, respectively. It was further discovered that the synthesized Mn(II)C was able to reduce the viscosity of oil sample 1 by 50.38%. The investigation revealed that the combination of Mn(II)C + K exhibited a significant synergistic catalytic impact on reducing viscosity. Initially, the viscosity reduction rate was 50.38%, which climbed to 61.02%. Subsequently, when catalyzed by the hydrogen supply agent isopropanol, the rate of viscosity reduction rose further to 91.22%. Several methods, such as freezing point analysis, thermogravimetric analysis, DSC analysis, component analysis, gas chromatography, wax crystal morphology analysis, and GC-MS analysis, were conducted on aqueous organic matter derived from heavy oil after undergoing different reaction systems. These analyses confirmed that the viscosity of the heavy oil was decreased. By studying the reaction mechanism of the model compound and analyzing the aqueous phase, the reaction largely involves depolymerization between macromolecules, breakdown of heteroatom chains, hydrogenation, ring opening, and other related consequences. These actions diminish the strength of the van der Waals force and hydrogen bond in the recombinant interval, impede the creation of a grid-like structure in heavy oil, and efficiently decrease its viscosity.

## 1. Introduction

**Figure 1:**
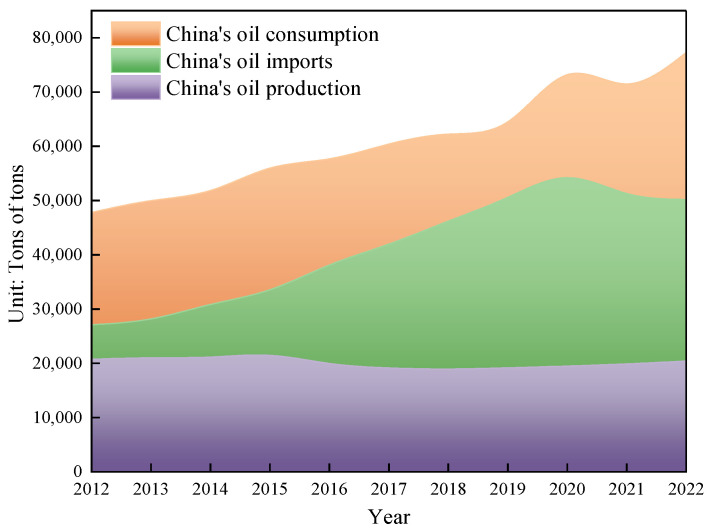
Changes in China’s oil production, consumption, and imports.

**Figure 2:**
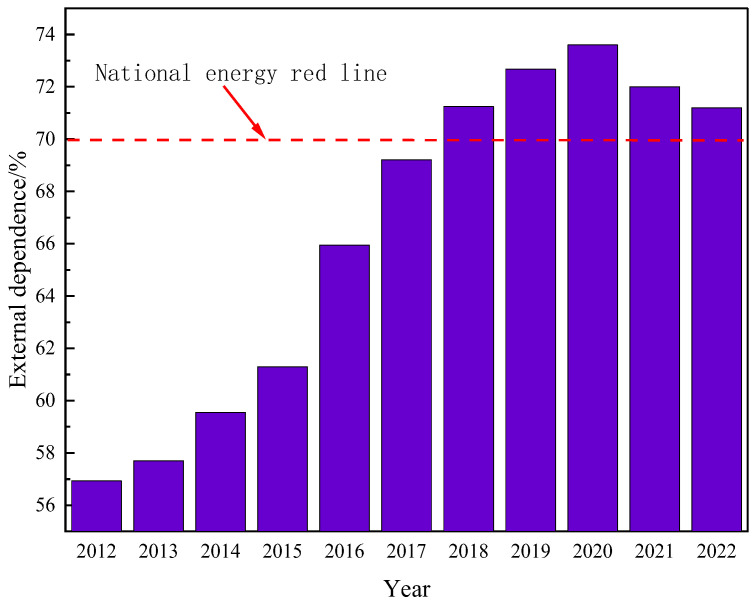
Changes in China’s dependence on foreign crude oil.

Due to the ongoing growth in the worldwide population and the continuing advancement of the social economy, there has been a substantial rise in the annual global demand for oil resources. Previously developed oil fields worldwide have been excessively exploited, resulting in a significant decline in crude oil production. The oil fields have often reached a stage of high water content, and there has been excessive consumption of the usual light crude oil. As a result, it has become challenging to meet the energy demands of humanity. According to the US Geological Survey, global oil demand is projected to rise by over 40% by 2025 due to economic and social progress. The world possesses a substantial amount of heavy oil resources, with an estimated geological reserve of roughly 815 billion tons, making up about 70% of the global oil reserves. These reserves are significantly larger than those of traditional crude oil [1]. The main countries with substantial heavy oil resources are Canada, Venezuela, Russia, the United States, and China. Hence, the retrieval of viscous crude oil holds significant significance [2]. China’s energy security will encounter unprecedented problems as a result of the restricted availability of conventional oil and the surging demand for oil. Given its status as the wealthiest developing nation globally, the Chinese government has prioritized the establishment of a reliable oil supply as a crucial national strategy to fulfill the growing demand for oil. China, with its booming economy, has become the largest importer of crude oil globally. As depicted in Figure 1, China’s oil imports have consistently grown, reaching a staggering 502 million tons by 2022. China’s reliance on imported crude oil is steadily growing, reaching 71.2% by 2022, as depicted in Figure 2. Hence, the exploration and extraction of non-traditional crude oil has been acknowledged as a significant and practical alternative for China to counterbalance the consequences of its diminishing conventional oil output and enhance its energy security [3,4].

China’s geological petroleum resources include a significant amount of heavy oil, making up approximately 25% of the total. This amounts to around 20 billion tons, with over 4 billion tons being recoverable [5,6]. China’s primary distribution of heavy oil is concentrated in the Shengli, Liaohe, Tahe, Tuha, and other oil fields [7].

Extracting heavy oil is highly challenging due to its intricate composition and the complicated structure of the deposit, which further complicates the extraction process [8,9], Heavy and extra-heavy oils possess a significant amount of gelatinous and asphaltene substances, which make the transportation and refining of oil more complex, resulting in higher costs for refining heavy oil products. Hydrothermal cracking technology is commonly employed to break chemical bonds in heavy oil molecules by using water as a catalyst at high temperatures. This process converts the recombinant parts into lighter components, reducing the content and viscosity of the heavy oil [10,11,12,13].

Water-soluble catalysts are one of the commonly used chemicals in the petroleum industry. Hot water is also a cheap and safe solvent for heavy oil; therefore, water-soluble catalysts were also the earliest catalysts used for crude oil cracking. Maity et al. [9] used water-soluble transition metal ruthenium (Ru) and iron (Fe) catalysts in their asphalt upgrading experiments, with desulfurization efficiencies of 21% and 18%, respectively. The first row of transition metals and Al^3+^ ions have significant catalytic activity towards thiophene and tetrahydrothiophene. These metal ions have the ability to convert larger molecules into smaller ones by cleaving the C-S link. Zhong [11] investigated the alterations in viscosity and molecular weight of Liaohe heavy oil when exposed to the catalytic effects of eight metal ions. The findings indicated that all the metal ions employed exhibited a distinct catalytic effect on reducing the viscosity of Liaohe heavy oil. Specifically, Fe, Co, and Mo are capable of reducing the viscosity of heavy oil by 60%, while the inclusion of the hydrogen donor naphthalene can achieve a viscosity reduction of over 90%. In addition, Chen et al. [14] studied the catalytic effect of a water-soluble transition metal complex on heavy oil in Yumen Oilfield, which reduced its viscosity by more than 70% at 180 °C. Research has shown that transition metal compounds have a significant catalytic effect on C-S bond cleavage. The complexation of metal atoms with organic sulfur is strengthened under the action of ligands, which break the C-S bond and initiates acid polymerization and a water–gas shift reaction, thus reducing the viscosity of heavy oil and improving the quality of heavy oil [15,16,17]. During the process of crude oil generation, different mineral components in the formation are most in contact and play an important role in catalytic viscosity reduction. The minerals in the reservoir are mainly composed of clay and non-clay minerals. High-carbon hydrocarbon molecules donate electrons to minerals on their surface due to the presence of Lewis acid, simultaneously creating free radicals. The rearrangement of free radicals promotes the fracture of C-C bonds and the generation of short-chain alkanes.

Therefore, in this paper, sodium citrate was used as a ligand to prepare a water-soluble catalyst to investigate the viscosity reduction effect of water-soluble catalysts and reservoir mineral catalysis on heavy oil and to investigate the viscosity reduction effect of reservoir minerals and water-soluble catalyst synergistic catalysis on heavy oil water pyrolysis. The objective was to combine the two methods in order to obtain a significant decrease in the viscosity of heavy oil at low temperatures.

## 2. Results and Discussion

### 2.1. Infrared Analysis

**Figure 3:**
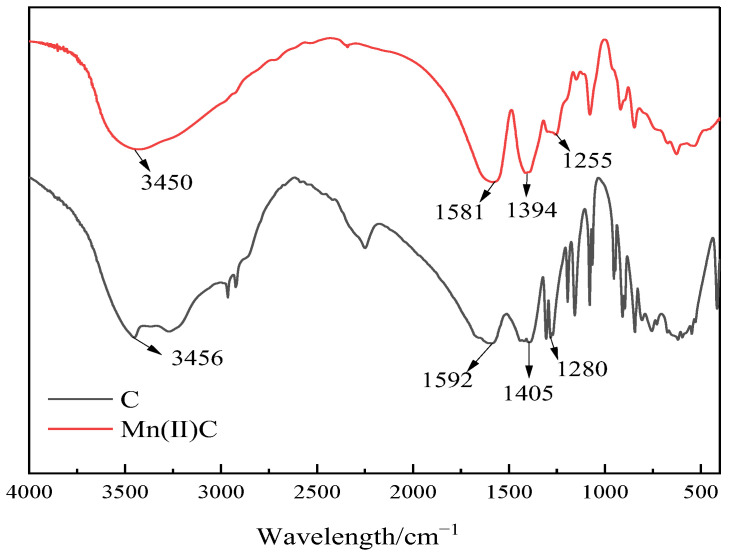
Infrared spectra of ligand C and catalyst Mn(II)C.

Infrared spectroscopy was used to evaluate the water-soluble catalyst Mn(II)C, as depicted in Figure 3. It can be seen from Figure 3 that the absorption peak of ligand C at 3456 cm^−1^ is the telescopic vibration peak of O-H, the strong absorption peak at 1592 cm^−1^ is the telescopic vibration peak of C=O in carboxylic acid, the absorption peak at 1405 cm^−1^ is the in-plane bending vibration peak of O-H, and the absorption peak at 1280 cm^−1^ is the telescopic vibration peak of C-O in carboxylic acid. Compared with ligand C, the peak position of Mn(II)C was blueshifted, mainly because the ligand formed a coordination bond with manganese ions.

### 2.2. Thermogravimetric Analysis

**Figure 4:**
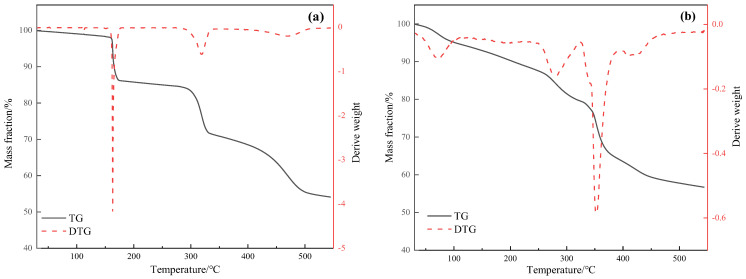
Thermogravimetry of ligand C (**a**) and water-soluble catalyst Mn(II)C (**b**).

Figure 4 shows the thermogravimetric differential thermal analysis (TG-DTA) curves of the catalysts Mn(II)C and ligand C. Thermal decomposition can be divided into the following stages. First, below 200 °C is the water loss stage, mainly organic matter precipitation, decomposition into water vapor and CO_2_. The mass loss of C this stage is about 14.24%, and the mass loss of Mn(II)C is about 9.70%. During the second stage, which occurred at temperatures of 200–350 °C, an exothermic peak was seen. This peak was mostly caused by the breakdown and oxidation of certain compounds. The mass loss during this stage, denoted as C, was measured to be 14.92%. Additionally, the mass loss of Mn(II)C was found to be 15.20%. Finally, at 350–450 °C, a strong exothermic peak appeared for Mn(II)C at 353 °C, the exothermic peak at 420 °C was exothermic carbon combustion, and the mass loss in this stage was about 15.71% [18,19,20]. Above 450 °C, the decomposition of both substances tends to be basically stable. Based on the data provided, it is evident that Mn(II)C was effectively produced and remained stable within the temperature range of hydrothermal cracking.

### 2.3. Changes in Viscosity

**Figure 5:**
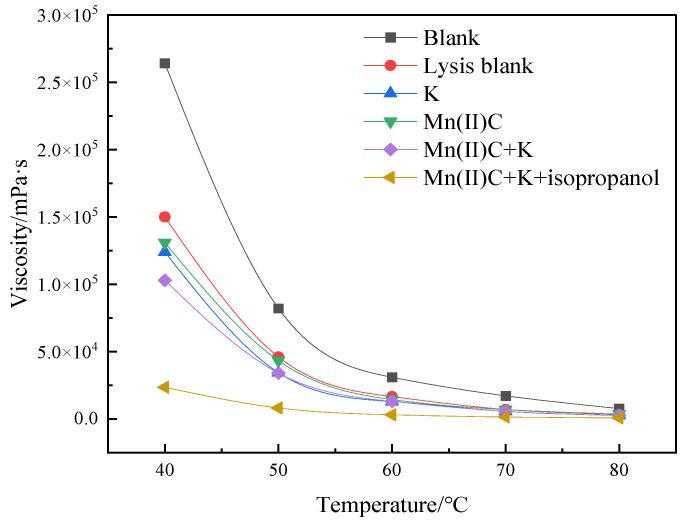
Viscosity–temperature curves of oil sample 1 under varying reaction circumstances, both before and after.

**Figure 6:**
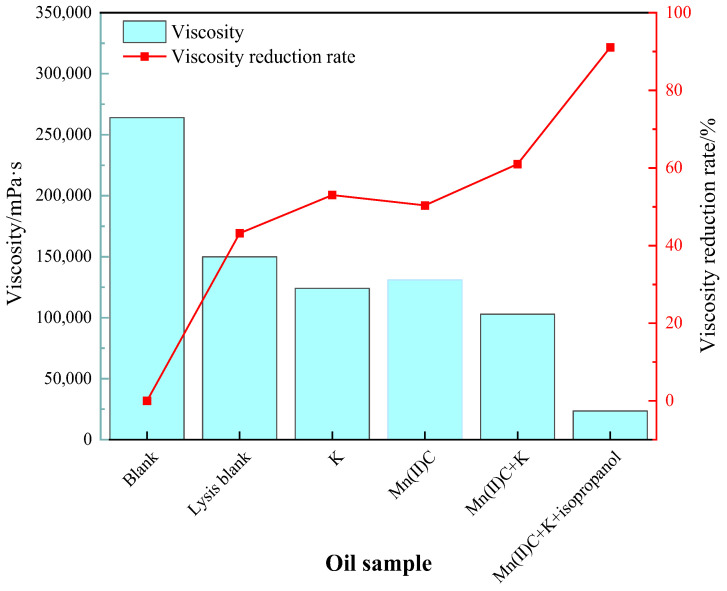
The impact of various reaction circumstances on the viscosity and the ability of oil sample 1 to reduce viscosity.

Figure 5 demonstrates that the viscosity reduction rate following the process of simple hydrothermal cracking of heavy oil was 43.18%. This finding highlights the significant contribution of water in both thermal cracking and viscosity reduction of heavy oil. The addition of reservoir minerals resulted in a 9.85% increase in the rate of viscosity reduction compared to the blank sample, suggesting that the presence of reservoir minerals promoted a decrease in viscosity of heavy oil. The water-soluble catalyst Mn(II)C exhibited a 7.20% increase in the rate of viscosity decrease compared to the cracking blank. Following the synergistic reaction between Mn(II)C and K, the reservoir minerals were able to catalyze by exchanging ions with the exogenous catalyst. This resulted in a 17.84% increase in the rate of viscosity reduction compared to the control group without cracking. Furthermore, the viscosity reduction effect was enhanced compared to the catalysis of Mn(II)C and the reservoir minerals separately. Figure 6 demonstrates that the catalysis of Mn(II)C + K + isopropanol resulted in a 41.12% increase in the rate of viscosity reduction compared to the cracking blank. The auxiliary isopropanol is added to the reaction system, because isopropanol itself has a diluting effect and can provide active hydrogen to the reaction system so that the free radicals after the broken bond are hydrogenated to generate small molecules, thereby ensuring stable reduction in the viscosity of heavy oil.

### 2.4. Assessment of the Stability and Universality of Catalysts in Reducing Viscosity

**Figure 7:**
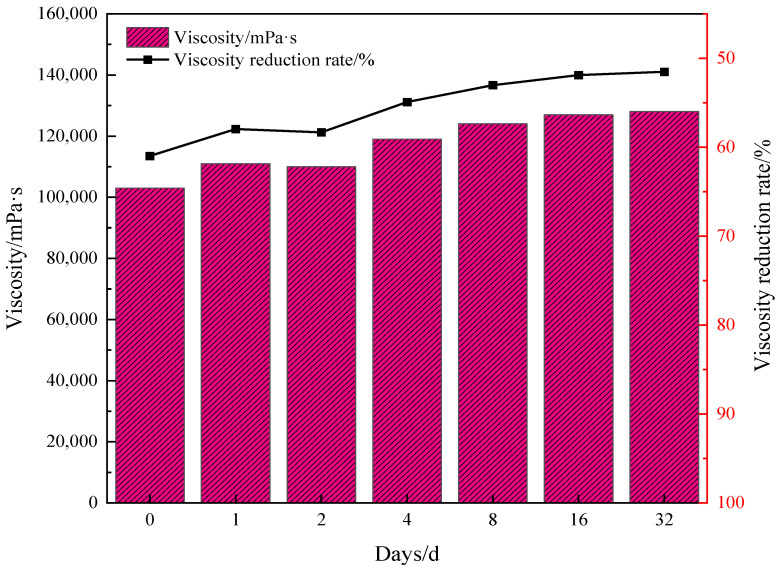
Assessment of the synergistic effect of Zn(II)O+K on the viscosity decrease of oil sample 1.

Under the conditions of reaction conditions of 180 °C for 4 h and water addition of 30%, the water-soluble catalyst and reservoir mineral addition were 0.2%. The viscosity–temperature properties of Mn(II)C + K were investigated after the reaction of oil sample 1 after 1–32 days, and the results are shown in Figure 7. It should be noted that the reaction conditions are optimized, and the results show that the viscosity remains basically constant after 4 h, so we determined that 4 h would work best. For the reaction temperature, when the temperature is lower than 180 °C, the hydrothermal cracking is not sufficient, and the viscosity drop is not the largest. When the temperature is higher than 180 °C, the structure of the catalyst may be damaged, which will affect its stability and catalytic effect. Therefore, the reaction conditions were finally determined to be 180 °C for 4 h. Otherwise stability, especially temperature, will be affected.

**Figure 8:**
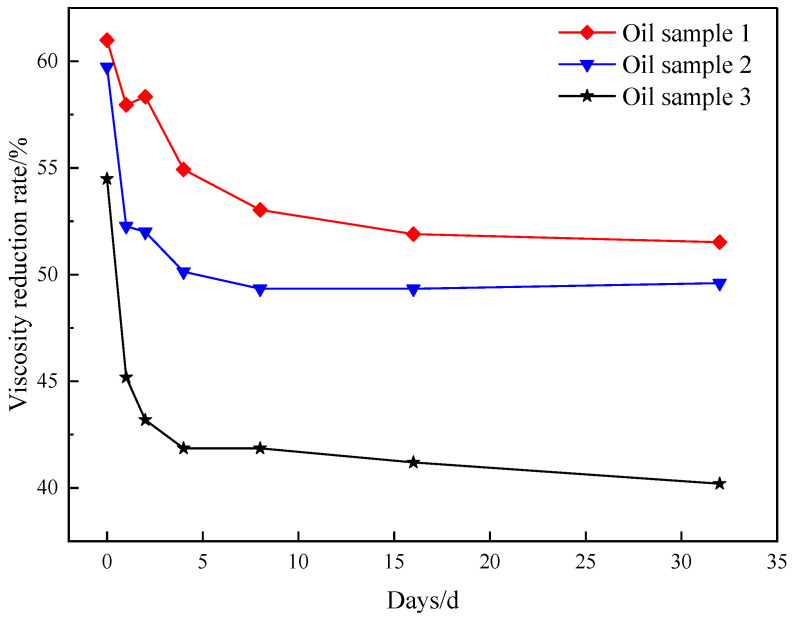
Evaluation of the synergistic effect of Zn(II)O+K universally on several oil samples.

Figure 7 demonstrates that following synergistic catalytic heavy oil water thermal cracking, the viscosity experiences a rapid rebound, with a faster rebound rate. Subsequently, the viscosity fluctuation range becomes smaller and the viscosity reduction rate stabilizes at approximately 51%. Based on the aforementioned analysis, it is evident that the synergistic catalytic stability of Mn(II)C + K is superior. Figure 8 illustrates the comprehensive assessment of Mn(II)C + K for various oil samples. The figure clearly demonstrates that the viscosity of different oil samples experiences a notable rebound within a brief timeframe. Subsequently, the viscosity reduction rate stabilizes at a consistent level. This phenomenon primarily arises from the presence of short-term heavy oil-containing heteroatom groups, which prompts the reformation of hydrogen bonds and other intermolecular forces. Consequently, the smaller molecules that were initially formed undergo a transformation into larger molecules. Moreover, the interconnection of alkyl chains leads to a rise in viscosity. After the intermolecular interaction reaches a stage of stability, the viscosity is reasonably consistent. The figure illustrates that the presence of Mn(II)C + K catalyst can result in a viscosity reduction rate of over 40% in the three oil samples. This suggests that the combined catalytic effect of Mn(II)C + K remains generally constant and reliable. Therefore, the results of this study can be generalized to the development of heavy oils with the same composition and structure. However, due to the strong pertinence of the thermal cracking of crude oil heavy oil, it may have different results for heavy oil with different compositions or different geological backgrounds, which is also a worldwide problem in this field.

### 2.5. Variations in Heavy Oil’s Pour Point Both before and after the Reaction

**Figure 9:**
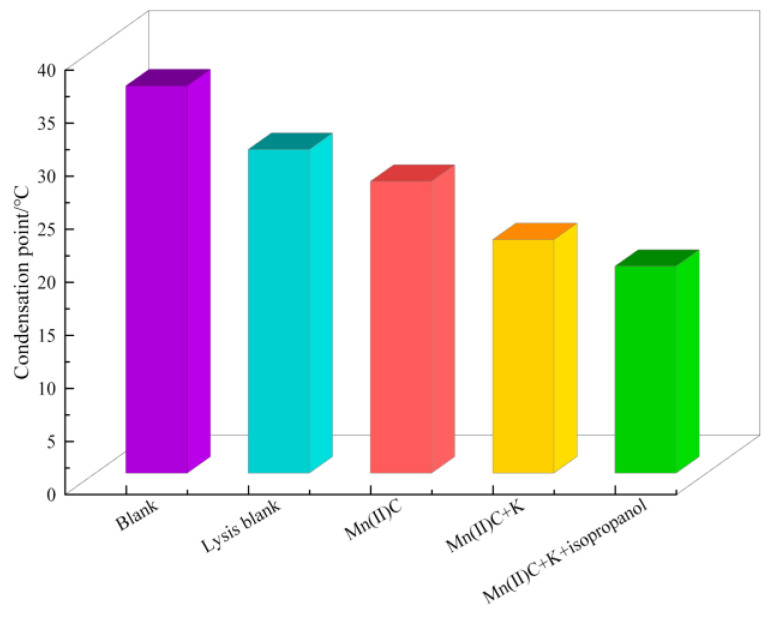
Comparison of solidification points of heavy oil.

Figure 9 clearly demonstrates that the initial oil sample has a notable pour point, which is substantially reduced after undergoing hydrothermal cracking. Specifically, the pour point lowers drastically from 36.5 °C to 30.5 °C, resulting in a remarkable decrease of 6 °C. The oil sample’s pour point was reduced to 22 °C and 8.5 °C through the synergistic catalysis of Mn(II)C + K. The condensation point is further decreased to 19.5 °C with the assistance of hydrogen supplied during the catalytic process of isopropanol.

### 2.6. Thermogravimetric Analysis of Heavy Oil before and after Reaction

**Figure 10:**
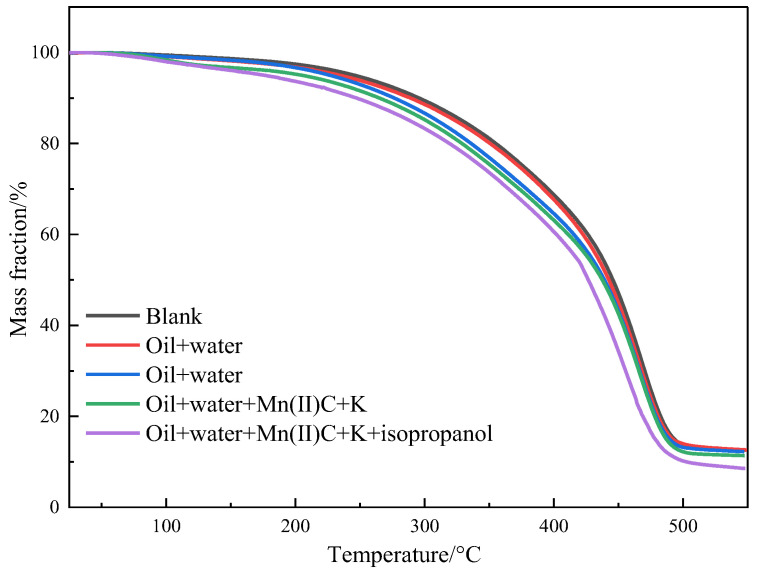
Thermogravimetric curves of oil sample 1.

Figure 10 illustrates the thermogravimetric analysis of oil sample 1 before and after the reaction in different reaction systems. The pyrolysis process of heavy oil can be classified into three distinct stages. In the first stage, which occurs at temperatures ranging from 25 to 150 °C, there is a volatilization of low-carbon hydrocarbons and water. The weight loss percentages for blank, oil + water, oil + water + K, oil + water + Mn(II)C + K, and oil + water + Mn(II)C + K + isopropanol are 2.58%, 3.19%, 3.33%, 4.77%, and 6.35%, respectively. During the second stage, which occurs at temperatures of 150–350 °C, the research indicates that the evaporation of light components and the disruption of weak chemical bonds result in a partial decomposition of specific asphaltenes. As a result, the oil samples experienced weight loss rates of 16.27%, 16.66%, 19.94%, 19.72%, and 20.06%, respectively. This indicates that a portion of the asphaltenes underwent hydrothermal cracking, resulting in the formation of low-carbon hydrocarbons. These low-carbon hydrocarbons then evaporated during thermogravimetric examination, causing an acceleration in the rate of weight loss. The temperature range of the third stage was 350 to 550 °C. The oil samples experienced weight loss rates of 68.50%, 67.55%, 64.50%, 64.10%, and 64.90%, respectively. This weight loss was mostly caused by the significant breaking of recombinant components in heavy oil. Based on the figure and analysis, the weight loss curves of several reaction systems clearly show a leftward shift in comparison to the blank oil sample. This observation provides additional evidence that recombination leads to the separation of certain lightweight constituents, hence decreasing the viscosity of heavy oil.

### 2.7. DSC Analysis

**Figure 11:**
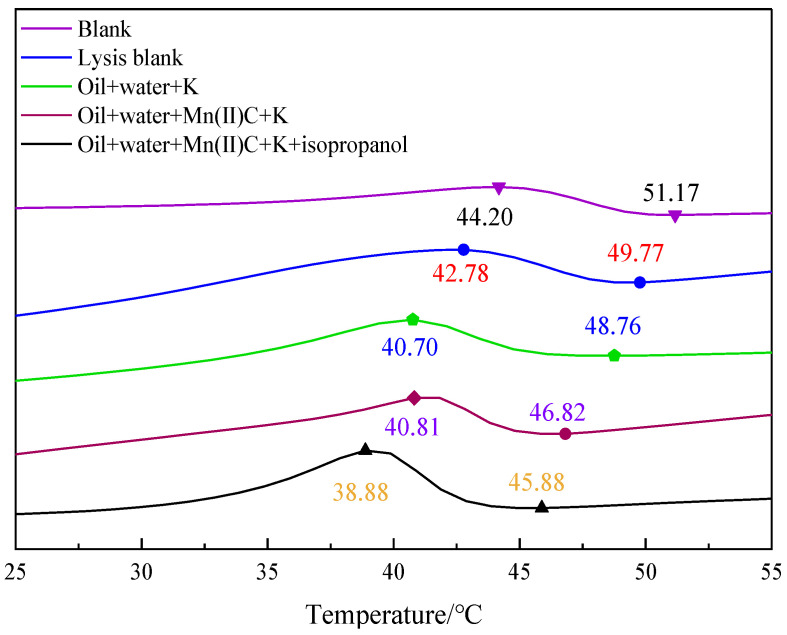
DSC curve of oil sample 1.

Figure 11 demonstrates that the wax precipitation point of oil sample 1 was 51.17 °C. After undergoing hydrothermal cracking, the wax precipitation point decreased to 49.77 °C. Additionally, the peak temperature of wax evolution decreased from 44.20 °C to 42.78 °C compared to the initial sample. When oil, water, and K were combined for the hydrothermal cracking reaction, the wax precipitation point further decreased to 48.76 °C. When it passed through oil + water + Mn(II)C + K synergistic reaction, the wax precipitation point was reduced to 46.82 °C, which was reduced by 4.35 °C compared with the wax precipitation point of the blank oil sample. Upon the introduction of isopropanol into the reaction system, the temperature at which wax precipitation occurred decreased to 45.88 °C and there was a notable shift in the highest temperature at which wax was produced, which now stood at 38.88 °C, which indicated that the hydrogen supply of isopropanol itself induced the formation of light components during heavy oil cracking, and at the same time, the dilutive dissolution of isopropanol itself inhibited the formation of wax deposition and reduced the peak value of wax evolution.

### 2.8. Elements and Four Components

**Figure 12:**
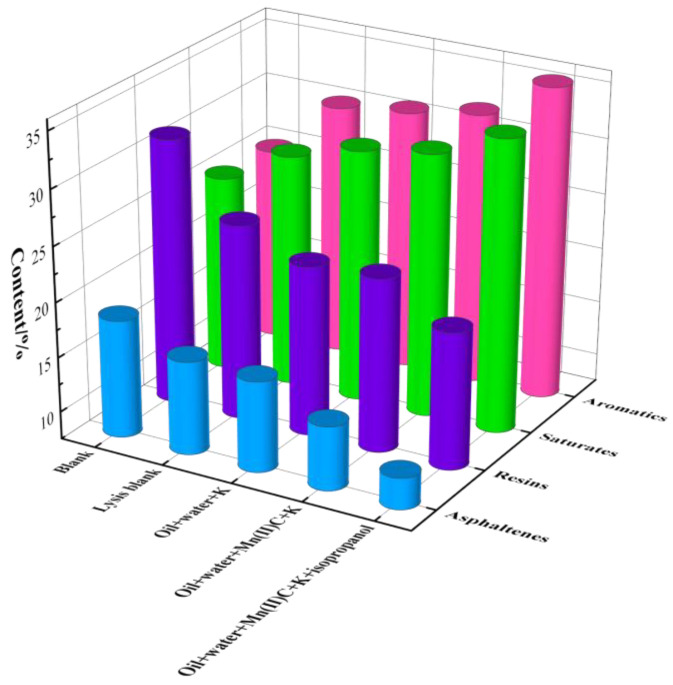
Component analysis of oil sample 1.

Figure 12 displays the results of the four-component analysis of oil samples following reaction under various reaction systems. In the absence of a catalyst, the levels of saturated hydrocarbons and aromatic hydrocarbons in the oil sample increased following the reaction, but the amounts of gum and asphaltene dropped. Mn(II)C + K synergistic catalysis exhibited excellent catalytic efficiency, resulting in the conversion of 31.31% saturated hydrocarbons, 32.11% aromatic hydrocarbons, 23.24% gums, and 13.34% asphaltenes in the oil samples following the reaction. Following the use of isopropanol-assisted catalysis to supply hydrogen, the oil sample had additional increases in its saturated hydrocarbon and aromatic hydrocarbon content, reaching 33.93% and 35.75%, respectively. The levels of gum and asphaltene reduced dramatically, with reductions of 19.95% and 10.37%, respectively. The data above show that the combined catalytic impact of external catalysts and reservoir minerals significantly increases the concentration of light components in heavy oil.

The elemental analysis results of oil samples obtained from different reaction systems are displayed in Table 1. The carbon-to-hydrogen ratios of the blank oil samples, oil samples catalyzed by Mn(II)C + K, and Mn(II)C + K + isopropanol catalyzed oil samples were 8.54, 8.35, and 8.33, respectively. It is evident that the C/H ratio decreased significantly, indicating that the catalyst facilitated the hydrogenation of unsaturated bonds [4]. The concentrations of carbon (C), nitrogen (N), and sulfur (S) in the oil samples declined to different extents after the catalytic reaction of Mn(II)C + K and Mn(II)C + K + isopropanol, while the concentration of hydrogen (H) increased. The reduction in viscosity of heavy oil can be ascribed to the fragmentation of C-X (X: N, S, O) bonds and the participation of water in the catalytic hydrothermal cracking procedure [7,21].

### 2.9. GC Analysis of Saturated Hydrocarbon Components

**Figure 13:**
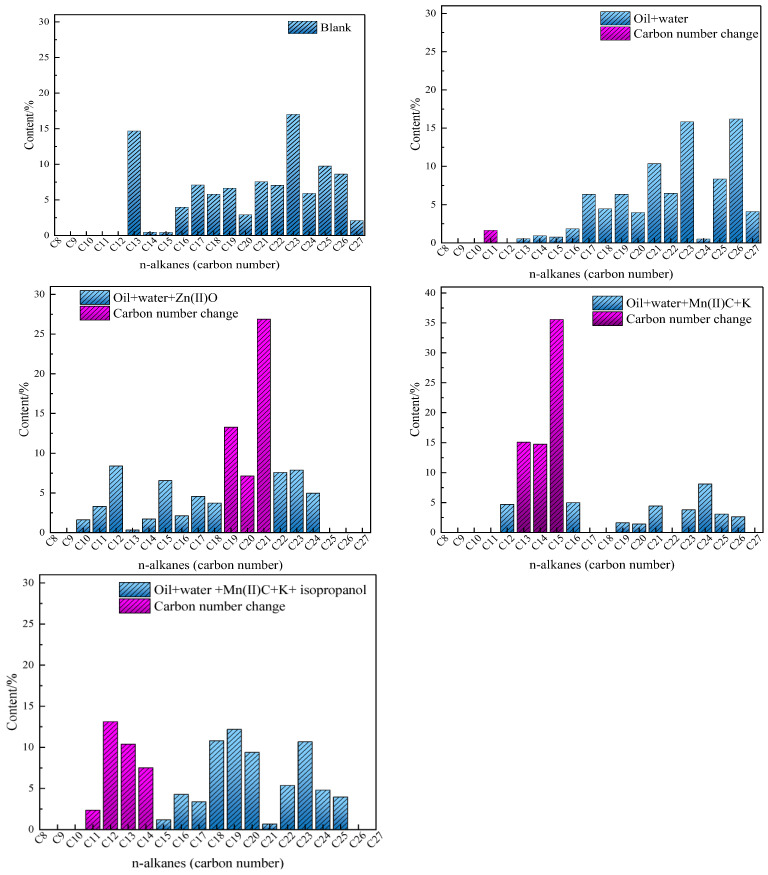
Carbon number distribution of oil samples.

The study examined the alterations in the distribution of carbon numbers in saturated hydrocarbons present in oil sample 1 following reactions with various catalytic systems. Gas chromatography (GC) was employed to assess the saturated hydrocarbons both before and after reactions. The chromatographic analysis findings are presented in Figure 13. The concentration of oil + water in the C11 and C12 fractions of the oil + water mixture rose significantly compared to the control sample in the higher carbon number range of the blank oil sample. The majority of carbon atoms in the mixture of oil, water, and potassium (K) are found in the range C15–C22. However, after the synergistic reaction, the distribution of carbon atoms in the mixture of oily oil, water, manganese(II) carbonate (Mn(II)C), and potassium (K) shifted dramatically towards a lower range: C12–C16. After adding isopropanol to the reaction system, the oil sample showed a significant reduction in high-carbon compounds, while the proportion of low-carbon compounds notably rose. Specifically, there was a considerable rise in the content of C12 and C13 compounds. The varying distributions of carbon numbers directly indicate the fluctuations in the amount of saturated hydrocarbons present in heavy oil. These changes in saturated hydrocarbon content have a direct impact on the freezing point of heavy oil, thereby demonstrating the alteration in freezing point observed in oil samples and the leftward-shift phenomenon observed in DSC thermal analysis results.

### 2.10. Wax Crystal Morphology

**Figure 14:**
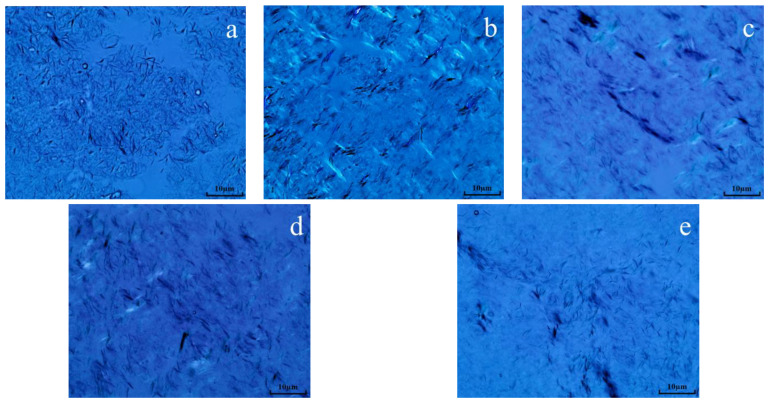
Wax crystal morphology of oil sample 1 ((**a**): blank; (**b**): cracking blank; (**c**): oil + water + K; (**d**): oil + water + Mn(II)C + K; (**e**): oil + water + Mn(II)C + K + isopropanol).

Polarizing microscopy was employed to examine the structure of wax crystals in oil sample 1 both prior to and during the reaction. Figure 14 demonstrates that the wax crystals in the oil samples exhibit a layered morphology resembling snowflakes prior to hydrothermal cracking. Following hydrothermal cracking, it became evident that the wax crystal accumulation structure was greatly diminished, with the majority of crystals forming needle-like accumulations. Following the reaction between oil, water, and K, the formation of wax crystals was much diminished, while the dispersion of wax crystals was enhanced. Following the synergistic catalytic reaction involving oil, water, Mn(II)C, and K, the dispersion of wax crystals became evident, and the shape of the wax crystals seemed more scattered. Following the reaction of oil, water, Mn(II)C, K, and isopropanol, the dispersion of the oil sample was further enhanced, with isopropanol acting as a solvent and diluent. The main mechanism that contributes to the improved scattering of wax crystals is the fragmentation of colloidal asphaltene macromolecules in heavy oil into hydrocarbons with low carbon content. These hydrocarbons function as solvents, resulting in a reduction in the freezing point and the point at which wax is produced following breaking. As a result, the arrangement of wax crystals becomes more spread out, which increases the flowability of thick oil [22,23].

### 2.11. GC-MS of Heavy Oil Aqueous Phase

**Figure 15:**
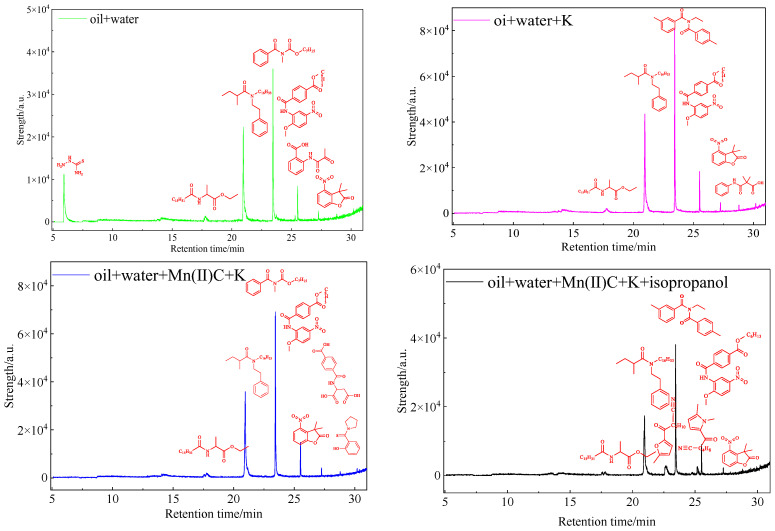
GC-MS analysis of polar substances dissolved in water after reaction of oil sample 1.

Figure 15 analysis reveals that hydrothermal cracking of the oil sample and catalytic reaction treatment of the catalyst result in the dissolution of polar organic compounds with heteroatoms in water. There has been a notable shift in the composition of polar compounds and chemicals containing heteroatoms in water, characterized by a rise in the diversity of heteroatomic substances. Hydrothermal cracking causes the rupture of chemical bonds in gum asphaltenes and other constituents of heavy oil, leading to a substantial decrease in viscosity. This is evident from the provided evidence. In addition, a small fraction of the heteroatomic organic matter that is generated when bonds are broken enters the composition of the aqueous phase.

## 3. Mechanism

### 3.1. Catalytic Aquathermolysis of Model Compounds

The catalytic hydrothermal cracking of heavy oil primarily entails the reaction between resin and asphaltene, which are large molecules composed of five-membered and six-membered rings with heteroatoms and alkyl branches. A set of model compounds with a unique chemical structure were chosen for catalytic hydrothermal breakdown research in order to investigate its mechanism for reducing viscosity. The seven model α-compounds were octene, phenol, thiophene, pyridine, quinoline, nonylphenol, and benzothiophene, and the liquid phase was isolated for further GC-MS analysis.

**Figure 16:**
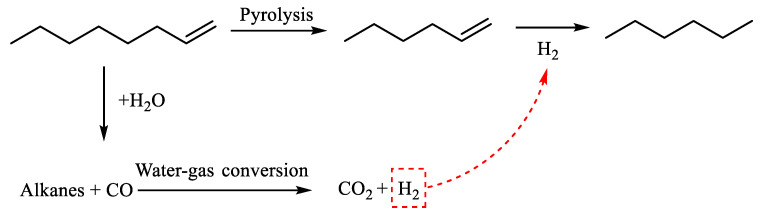
α-Octene reaction mechanism.

Table 2 demonstrates that n-hexene was produced 2.761 min after the reaction of α-octene. Octene experienced cracking during the reaction, resulting in the production of low-carbon olefins, such as n-hexene. The shorter-chain alkanes that were released underwent hydrothermal cracking and were converted into water gas. This further reaction resulted in the generation of CO_2_ and H_2_. At a time interval of 3.094 min, the compound n-hexane was produced. Furthermore, the preceding reaction involving the hydrogenation of n-hexane resulted in the generation of additional n-hexane. The reaction path of α-octene cleavage was determined by combining the results of GC-MS analysis with information from the literature [24]. This is illustrated in Figure 16.

**Figure 17:**
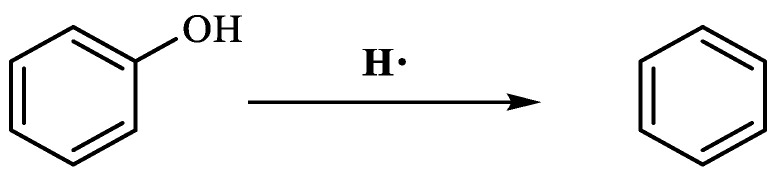
Phenol reaction mechanism.

Table 3 demonstrates that benzene was produced 5.860 min after the reaction of phenol. This occurred under conditions of elevated temperature and abundant hydrogen supply. The reduction in phenol hydroxyl resulted in the formation of water, which diminished the presence of hydrogen bonding between molecules. Consequently, the viscosity of heavy oil was decreased. Based on the results of GC-MS analysis and combined with the literature [25,26], the reaction path of lysis was derived, as shown in Figure 17.

**Figure 18:**
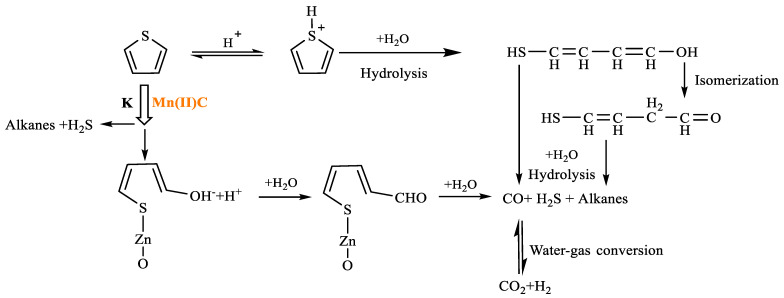
Thiophene reaction mechanism.

As shown in Table 4, cyclohexane was formed at 5.465 min after thiophene reaction, and may have occurred by hydrogenation of solvent benzene and H^+^ reaction in the reaction system. Phenol was formed at 13.088 min by redox reaction between solvent benzene and CO and H_2_ in the reaction system. At 6.451 min, m-dimethylcyclohexane was formed by a rearranging reaction of low-carbon hydrocarbons. The chemical mechanism for the decomposition of thiophene was established by analyzing the results obtained from gas chromatography–mass spectrometry (GC-MS) and a comprehensive examination of the existing scientific literature [27,28,29,30]. This pathway is illustrated in Figure 18.

**Figure 19:**
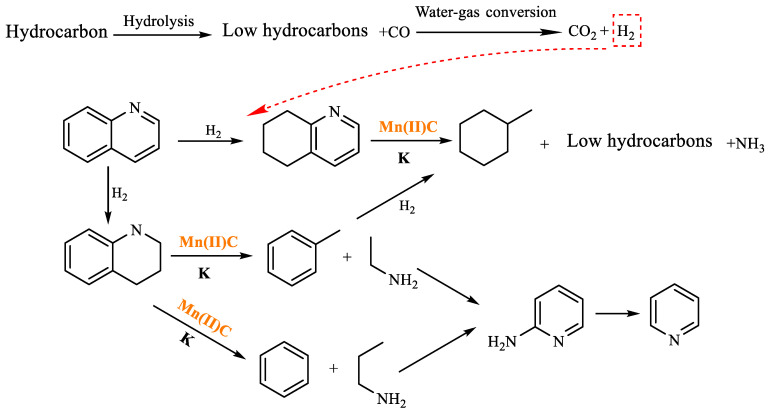
Quinoline reaction mechanism.

As shown in Table 5, methylcyclohexane was formed in the reaction product at 4.725 min, the benzene ring in quinoline was hydrogenated, and the transition metal cation coordinated with the nitrogen atom such that the C-N bond was broken. Methylcyclohexane was further generated after ring opening, recombination, and hydrogenation in the system. Toluene was formed at 5.900 min, and after the pyridine ring was hydrogenated in quinoline, toluene was generated by intramolecular ring opening and hydrogenation under the action of high temperature, catalyst, and additives. At 5.190 min, pyridine is generated, which is formed by cyclized deamination of ethylamine and propylamine during cleavage. The hydrothermal cracking reaction mechanism of quinoline was determined by analyzing the findings of GC-MS analysis and reviewing relevant literature [31,32], as depicted in Figure 19.

**Figure 20:**
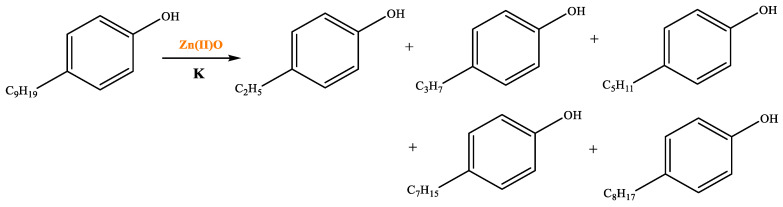
Reaction mechanism of nonylphenol.

Table 6 demonstrates that there is a higher number of peaks occurring at the 26 min mark, which are distinctive peaks associated with nonylphenol. Nonylphenol primarily undergoes the breaking and reforming of carbon–carbon bonds under diverse reaction circumstances, resulting in the formation of various chemicals. During hydrothermal processes, the shorter alkyl chains separate and mix with OH- to produce alcohols. At elevated temperatures, these alcohols oxidize and produce CO_2_. The hydrothermal cracking of nonylphenol was developed using the findings from GC-MS analysis and in combination with the existing literature [18,33], as depicted in Figure 20.

Table 7 demonstrates that cyclohexane was produced 2.761 min following the reaction of benzothiophene. Furthermore, cyclohexane was synthesized through catalytic thermal cracking, hydrodesulfurization, and catalytic hydrogenation. Toluene was synthesized at a reaction time of 5.600 min. It was previously shown in the literature that o-methylphenol underwent hydrogenation and deoxygenation to produce toluene [34,35,36]. The mechanism of the hydrothermal cracking reaction of benzothiophene was determined by integrating the findings of GC-MS analysis with relevant information from literary sources. It should be noted that this model is based on a limited number of model compounds, so this mechanism also takes into account all compounds as much as possible. However, the properties of heavy oil in different oil fields and wells are very different, so the model also has its limitations.

### 3.2. Catalytic Mechanism

The mechanism can be separated into the following stages.

(1) The abundance of glial asphaltene in heavy oil leads to a pronounced van der Waals force between the layers, causing the units to stick together. This phenomenon is visually observed as high viscosity and limited fluidity. The introduction of external catalysts has a significant impact on the active site, leading to both partial and permanent depolymerization as well as partial and loose binding. Consequently, certain unstable units undergo depolymerization and separation, leading to a substantial decrease in the viscosity of heavy oil.
(2) C-S, C-O, and C-N bonds separate as a result of interactions between the external catalyst and the heteroatoms in the recombination component, which breaks the hydrogen bonds between some high carbon hydrocarbon molecules.
(3) Reservoir minerals have a negatively charged surface as a result of lattice substitution, which allows them to absorb cations. This characteristic allows minerals in reservoirs to function as efficient catalysts and transporters. The transition metals found in the catalyst from another source can easily substitute sodium/calcium ions in the clay, thereby becoming the active sites in the process. Transition metals possess several vacant orbitals, allowing them to readily engage with electron-rich compounds found in heavy oil. This interaction significantly enhances the catalytic efficiency of hydrothermal cracking [37].
(4) At elevated temperatures, clay minerals exhibit strong acidic properties. The catalytic mechanism by which the mineral matrix produces oil and gas involves the formation of carbonium ions. Specifically, the acid centers on the surface of the mineral matrix facilitate the conversion of kerogen into carbonium ions. The catalytic action is accomplished by decomposing and transferring carbonium ions [38,39]. Non-clay minerals such as quartz and calcite have the ability to absorb free cations and create L-acid, which promotes the transformation of kerogen [40,41]. The existence of Lewis acid on mineral surfaces enhances the electron donation by high-carbon hydrocarbon molecules, resulting in the formation of free radicals. These free radicals then undergo rearrangement and encourage the breaking of C-C bonds, resulting in the formation of short-chain alkanes. Clay minerals function as a Brønsted acid by supplying a proton (H^+^) to adsorbed organic molecules. The proton (H^+^) is generated through the dissociation of water molecules that are adsorbed and present in the interlayer, along with exchangeable cations. This process mostly involves the formation of transition-state carbonium ions [42,43].
(5) Water molecules adhere to the surface of clay particles by adsorption. This phenomenon arises due to the high electron affinity of L-acid, which enables it to form a covalent bond by sharing a pair of electrons with the hydroxyl group in water. Consequently, the hydroxyl group gets strongly bonded to the surface of L-acid, while the remaining H^+^ ion is readily released. This process converts L-acid into B-acid. When clay minerals lose water molecules, as a result of proton deficiency, B-acid sites undergo a progressive transformation into L-acid sites [44,45,46]. The presence of clay minerals in this reaction system increases the reactivity of water/steam, reduces the energy needed for the reaction to take place, accelerates the disruption of hydrogen bonds in high-carbon hydrocarbon compounds, and improves the ability to decrease the viscosity of heavy oil.

## 4. Materials and Methods

### 4.1. Materials

The experiment utilized oil samples obtained from three different oil fields: Henan Oilfield in Henan Province, China (referred to as oil sample 1), Henan Nanyang Oilfield Henan Province, China (referred to as oil sample 2), and Henan Tanghe Oilfield Henan Province, China (referred to as oil sample 3). Table 8 displays the characteristics of heavy oils. The reagents used in the experiment are highly pure and do not require any additional treatment.

### 4.2. Preparation of Water-Soluble Exogenous Catalysts

**Figure 21:**
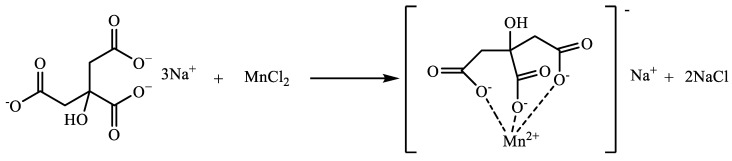
Reaction mechanism of Mn(II)C.

Sodium citrate (C) was used as the ligand in this experiment for the synthesis of a water-soluble catalyst, and manganese chloride and ligand sodium citrate were dissolved in water at a molar ratio of 1:1 and heated and refluxed for 4 h. The complex was named Mn(II)C, and the reaction mechanism is shown in Figure 21.

### 4.3. Water Thermal Cracking

**Figure 22:**
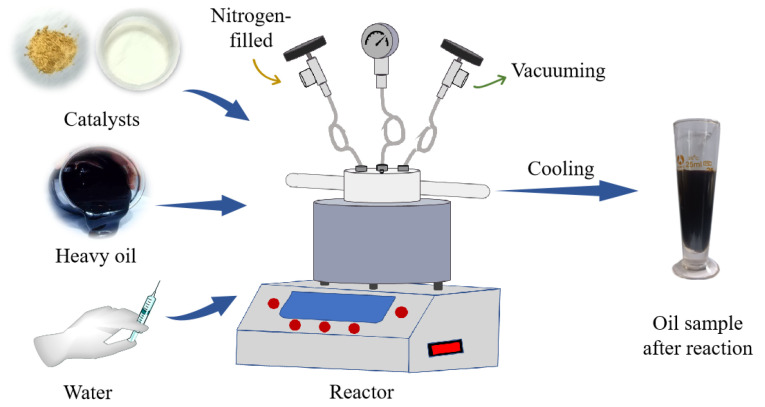
Catalyst catalyzes the thermal cracking process of heavy oil water.

Take a specific quantity of oil sample and place it in a water bath at a temperature of 65 °C. After maintaining a constant temperature for 1 h, transfer 30 g of the oil to the reactor. Next, introduce the prepped catalyst into the reactor at a proportion of 0.2% relative to the weight of the oil. Create a vacuum and fill the reactor with nitrogen. Allow the reaction to occur at a temperature of 180 °C for 4 h. Then, add a specific amount of water based on a water–oil mass ratio of 30%. Add the catalyst at a ratio of 0.2% to the mass of the oil. After the reaction has finished, lower its temperature to match that of the surrounding room, then separate it using a centrifuge. Then, transfer the separated substance into a measuring cup and proceed to measure its temperature, viscosity, and other physical characteristics. Figure 22 illustrates the reaction process.

### 4.4. Characterization

The viscosity of heavy oils is measured [47]. The rate of decrease in viscosity of the oil, Δη%, was calculated using the formula ((η0 − η)/η0) × 100, where η0 and η (mPa∙s) represent the initial and final viscosities of the oil, respectively [14,15]. In addition, the heavy oil components were analyzed following the guidelines of China Petroleum Industry Standard [48]. The elemental compositions (carbon, hydrogen, nitrogen, and sulfur) of the initial oil and enhanced oil were determined using the Elementar Vario EL Cube. An examination of the four components of petroleum asphaltene was carried out following the guidelines of standard [49]. Thermogravimetric analysis was used to evaluate the distribution of carbon numbers in crude oil across different temperature ranges. The oil samples were subjected to heating in a nitrogen atmosphere from 30 °C to 550 °C at a rate of 10 °C per minute. The wax precipitation point of heavy oil was tested according to the standard [50]. The examination of heavy oil using different scanning calorimetry (DSC) techniques was conducted using the a Mettler-Toledo DSC822e DSC apparatus (Mettler-Toledo Instruments Co., Ltd., Greifensee, Switzerland). The studies were conducted in a nitrogen atmosphere with a flow rate of 50 mL/min and a temperature range of −25 to 50 °C. The wax crystals in the crude oil were analyzed by examining the microstructure using saturated hydrocarbons. The examination was conducted at a temperature of 15 °C (±0.2 °C) using a polarizing microscope (BX41-Olympus, Tokyo, Japan). The composition analysis of the model compound was performed using gas chromatography–mass spectrometry (GC-MS) with a 7890A-5975C apparatus located in Santa Clara, CA, USA. The carrier gas employed was hydrogen, with a constant flow rate of 25 mL/min. The composition analysis of the samples was conducted using the DRS chemical database.

## 5. Conclusions

The synergistic catalytic effect of oil-soluble exogenous catalysts and reservoir minerals in hydrothermal cracking of heavy oils was studied. Mn(II)C + K has the best viscosity reduction effect. Compared with the blank of oil sample 1 at 40 °C, the viscosity reduction rate can reach 61.02%, and compared with the cracked blank, the viscosity reduction rate can reach 31.40%. After isopropanol catalysis, the viscosity reduction rate is further increased by 41.12% to 91.2% compared with the cracked blank, thereby reducing the viscosity of the heavy oil. After the hydrothermal decomposition of heavy oil, the thermogravimetric and DSC analysis results showed that the recombinant fraction decreased and the content of light components increased. In addition, the study can be applied to the heavy oil in selected oil fields on a large scale, but the biggest challenge is the effect of temperature. Because the temperature is too low to produce the catalytic effect, too high a temperature will damage the catalyst structure and cause the catalytic effect to fail.

## Figures and Tables

**Table 1:** Element analysis results of oil samples.

Oil Sample	C/%	H/%	N/%	S/%	C/H
Blank	86.22	10.10	2.25	0.45	8.54
Lysis blank	85.08	10.06	2.17	0.39	8.46
Oil + water + K	84.11	10.01	2.15	0.35	8.40
Oil + water + Mn(Ⅱ)C + K	84.21	10.09	1.91	0.33	8.35
Oil + water + Mn(Ⅱ)C + K + isopropanol	84.13	10.10	1.58	0.30	8.33

**Table 2:** α-Compound after octene reaction.

Compound Structural Formula α-Octene + Water	Compound Structural Formula α-Octene + Water + K	Compound Structural Formula α-Octene + Water + Mn(Ⅱ)C + K	Compound Structural Formula α-Octene + Water+ Mn(Ⅱ)C + K+ Isopropanol
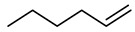			
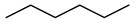			
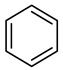			
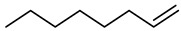			

**Table 3:** Compounds after phenol reaction.

Compound Structural Formula Phenol + Water	Compound Structural Formula Phenol + Water + K	Compound Structural Formula Phenol + Water + Mn(Ⅱ)C + K	Compound Structural Formula Phenol + Water + Mn(Ⅱ)C + K + Isopropanol
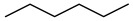			
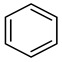			
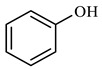			

**Table 4:** Compounds after thiophene reaction.

Compound Structural Formula Thiophene + Water	Compound Structural Formula Thiophene + Water + K	Compound Structural Formula Thiophene + Water + Mn(Ⅱ)C +K	Compound Structural Formula Thiophene + Water + Mn(Ⅱ)C + K+ Isopropanol
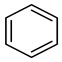			
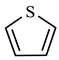			
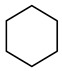			
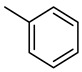			
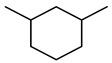			
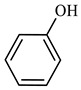			

**Table 5:** Compounds after quinoline reaction.

Compound Structural Formula Quinoline + Water	Compound Structural Formula Quinoline + Water + K	Compound Structural Formula Quinoline + Water + Mn(II)C + K	Compound Structural Formula Quinoline + Water + Mn(Ⅱ)C + K + Isopropanol
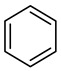			
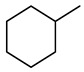			
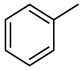			
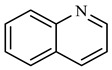			

**Table 6:** Compounds after nonylphenol reaction.

Compound Structural Formula Nonylphenol + Water	Compound Structural Formula Nonylphenol + Water + K	Compound Structural Formula Nonylphenol + Water + Mn(Ⅱ)C + K	Compound Structural Formula Nonylphenol + Water + Mn(Ⅱ)C + K + Isopropanol
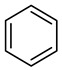			
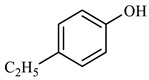			
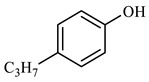			
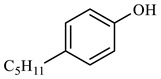			
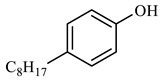			
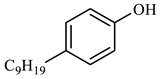			
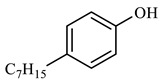			

**Table 7:** Compounds after benzothiophene reaction.

Compound Structural Formula Benzothiophene + Water	Compound Structural Formula Benzothiophene + Water + K	Compound Structural Formula Benzothiophene + Water + Mn(Ⅱ)C + K	Compound Structural Formula Benzothiophene + Water + Mn(Ⅱ)C + K + Isopropanol
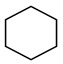			
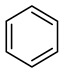			
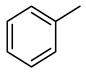			
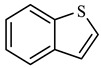			

**Table 8:** Main physical parameters of heavy oil.

Heavy Oil	Pour Point (°C)	Water Content (%)	Saturates (%)	Aromatics (%)	Resins (%)	Asphaltenes (%)
Oil sample 1	38.0	15.5	25.26	33.98	25.55	15.21
Oil sample 2	20.0	17.0	31.16	28.73	16.67	23.44
Oil sample 3	19.6	12.5	24.76	31.28	18.57	25.39

## Data Availability

The data presented in this study are available wholly within the manuscript.
